# Associations of serum concentrations of metal nutrients with postpartum anemia among pregnant Chinese women: A large retrospective cohort study

**DOI:** 10.3389/fnut.2023.1086082

**Published:** 2023-04-17

**Authors:** Geng-dong Chen, Ting-ting Pang, Peng-sheng Li, Zi-xing Zhou, Xiao-yan Gou, Hai-yan Wang, Dong-xin Lin, Da-zhi Fan, Hong-li Li, Zheng-ping Liu

**Affiliations:** ^1^Department of Obstetrics, Foshan Institute of Fetal Medicine, Affiliated Foshan Maternity & Child Healthcare Hospital, Southern Medical University, Foshan, Guangdong, China; ^2^Department of Medical Records, Affiliated Foshan Maternity & Child Healthcare Hospital, Southern Medical University, Foshan, Guangdong, China; ^3^Department of Obstetrics, Affiliated Foshan Maternity & Child Healthcare Hospital, Southern Medical University, Foshan, Guangdong, China

**Keywords:** metal concentrations, postpartum anemia, pregnant women, Chinese, retrospective cohort study

## Abstract

**Background and Aims:**

The association between serum concentrations of metal nutrients in pregnancy and postpartum anemia has not been widely studied. This study aimed to determine this association in a large retrospective cohort study.

**Methods:**

We included 14,829 Chinese women with singleton pregnancies. Serum concentrations of metals before 28 weeks of gestation, the occurrence of postpartum anemia and other potential covariates were obtained from their laboratory or medical records. Cox regression and restricted cubic spline regression models were used to explore the relationship between serum concentrations of metal nutrients in pregnancy and postpartum anemia.

**Results:**

After adjustment for covariates, higher concentrations of iron (Fe), magnesium (Mg) and zinc (Zn) and lower concentrations of copper (Cu) were associated with a lower risk of postpartum anemia. Compared with those whose serum concentrations of metal nutrients were in the bottom quintile (Q1), the hazard ratios (HRs) of those whose serum concentrations of metal nutrients were in the top quintile (Q5) were 0.57 (95% confidence interval (CI): 0.50, 0.64) for Fe, 0.67 (95% CI: 0.60, 0.76) for Mg, 0.82 (95% CI: 0.73, 0.93) for Zn, and 1.44 (95% CI: 1.28, 1.63) for Cu. L-shaped curve relationships were found between increasing concentrations of Fe, Mg, and Zn and incidence of postpartum anemia. Higher serum concentrations of Cu were associated with an increased risk of postpartum anemia. Serum concentrations of Fe in Q5 were associated with a lower risk of postpartum anemia when they coincided with serum concentrations of Mg in Q5, Zn in Q5, or Cu in Q1.

**Conclusion:**

Higher serum concentrations of Fe, Mg, and Zn, and lower serum concentrations of Cu were associated with a lower risk of postpartum anemia among pregnant women.

## Introduction

Postpartum anemia is a major and persistent public health problem worldwide. Its prevalence is high in developed countries (22–50%) and even higher in developing countries (50–80%) (1). Compared with women without postpartum anemia, those with postpartum anemia have higher risks of having depression (2, 3), fatigue and providing less responsive care for their babies (2). In addition, women with postpartum anemia due to acute blood loss have higher risks of sepsis (4) and mortality (5).

Oral iron (Fe), intravenous (IV) Fe and blood transfusion are common treatments for postpartum anemia (6). However, challenges remain in the use of these treatments and no optimal treatment has been identified. Up to 40% of women exhibit intolerance for oral Fe, in the form of adverse gastrointestinal effects (7). Compared with oral Fe, IV Fe is more effective in treating anemia (8–10), but Danish guidelines recommend against using IV Fe due to its having more severe side effects, a higher cost and there being a lack of sufficient evidence for its clinical efficacy (6). Compared with oral and IV Fe, blood transfusion is more expensive and puts women at higher risk of exposure to infectious pathogens. Given these treatment challenges, it is critical to identify interventions that can effectively and safely prevent postpartum anemia.

Fe is an essential nutrient for maintaining normal hemoglobin concentrations and Fe supplements are commonly used to prevent anemia (11). However, Fe overload or over supplementation can lead to increased adverse outcomes, such as gestational diabetes mellitus (GDM) (12). It is therefore crucial to determine optimal concentrations and supplemental doses of Fe to prevent postpartum anemia without causing adverse effects.

Deficiencies of several other metal nutrients, such as zinc (Zn), magnesium (Mg) and copper (Cu), may also be associated with anemia (13–15). In addition, deficiencies of these metal nutrients increase the risk of anemia in children, adolescents, adults and older adults (14, 16, 17). However, there has been little examination of associations between concentrations of metal nutrients during pregnancy and postpartum anemia, and some of these non-Fe metal nutrients (e.g., Zn and Cu) may interact with Fe metabolism and affect the development of anemia (13, 18, 19). Thus, the combined correction of these trace element deficiencies may represent a viable intervention for the prevention of anemia.

This retrospective cohort study aimed to explore the associations and dose–response relationships of maternal concentrations of seven metal nutrients during pregnancy (measured before 28 weeks of gestation) with the risk of postpartum anemia in Chinese women aged 18 to 45 years old. This study also investigated the potential combined influence of serum concentrations of Fe and other metal nutrients on the risk of postpartum anemia.

## Material and Methods

### Participants

This retrospective cohort study was carried out at a large obstetrics hospital in Foshan, Guangdong province, China. We reviewed the obstetric medical records from March 1, 2015 to July 31, 2018, of 16,930 women aged 18 to 45 years who had delivered their babies at the hospital and whose serum concentrations of metals (Fe, Mg, Cu, Zn, calcium [Ca], lead [Pb] and manganese [Mn]) were measured during pregnancy. Then, 2,101 women were excluded on the basis of their having (a) incomplete outcome data or exposure indicators (1,146 women); (b) multiple pregnancy; (c) a history of abnormal placental implantation (24 women) or placenta previa (147 women); (d) a baseline serum concentration of Fe < 6.5 μmol/l (246 women); or (e) serum concentrations of metals measured after 28 weeks of gestation (558 women). This yielded 14,829 women for inclusion in this study. The study protocol was approved by the ethics committee of the Affiliated Foshan Maternity and Child Healthcare Hospital, Southern Medical University. The Affiliated Foshan Maternity and Child Healthcare Hospital provided administrative permission for the research team to access and use the data.

### Measurement of serum concentrations of metals

Serum concentrations of metals were measured during the women’s’ regular obstetric check-ups in the hospital. Only women whose serum concentrations of metals were measured before 28 weeks of gestation were included, as this ensured that only true and early associations between serum concentrations of metals in pregnancy and postpartum anemia were obtained. Blood samples were collected by nurses in the clinic and then transported to the laboratory within 1 h for measurement. Serum concentrations of metals were measured using the polarography method (AS-9000\u00B0C, AWSA, Wuhan, China), with a detection limit of less than or equal to 1 × 10^−8^ mol/l. The coefficient of variation and the relative error of the detection was less than or equal to 1% during daily quality control.

### Postpartum anemia and other covariates

Two staff members (G.D.C and T.T.P) independently extracted outcome data from medical records. In this retrospective study, the outcomes of postpartum anemia were collected from medical records and based on the ICD-10 code of O99.001. The diagnosis of postpartum anemia was made by professional doctors according to the criteria of the concentration of hemoglobin in their peripheral blood was less than 110 g/l happened postpartum, but not necessarily happened at 1 week postpartum. Other covariates reviewed and extracted from medical records were age, body mass index (BMI) at delivery, gestational week of delivery, parity, delivery mode, occurrence of abnormal placental implantation, placenta previa, immediate postpartum hemorrhage, and intrapartum injury.

### Statistical analysis

Continuous variables are reported as means ± standard deviations (SDs) or medians (interquartile ranges). Outcome variables (serum concentrations of metals) are reported as medians with interquartile ranges.

The women were divided into quintiles according to their serum concentrations of metals, with the bottom quintile (Q1) comprising those with the lowest serum concentrations of metals and the top quintile (Q5) comprising those with the highest serum concentrations of metals. Cox regression analyses were used to determine the associations between serum concentrations of metals and postpartum anemia. Follow-up time was calculated as the time between measurement of serum concentrations of metals and discharge after delivery. Two models were used in the analysis: a univariate model (Model 1) and a multivariate model (Model 2) adjusting for potential covariates (age, gestational week of delivery, BMI, parity, delivery mode, immediate postpartum hemorrhage and intrapartum injury). Those in Q1 were used as the reference group for most metals (Fe, Mg, Cu Zn, Ca and Mn). For inverse Cu and Pb, those in Q1 had the highest serum concentrations and those in Q5 had the lowest serum concentrations. Restricted cubic spline regression with five knots was performed to explore the dose–response associations between serum concentrations of metals and postpartum anemia. Likelihood ratio tests were used to test for nonlinearity of these associations. Analyses were performed using SPSS 20.0 software (Chicago, IL, USA) and R statistics software version 3.6.3.^1^ Plots were also drawn using R 3.6.3 software. Results with a two-sided *p* value of less than 0.05 were considered statistically significant.

## Results

This retrospective cohort study included 14,829 women with singleton pregnancies and who were aged 29.9 ± 4.80 years old. They had delivered their babies at a mean gestational age of 38.9 ± 1.76 weeks and had a mean parity of 1.41 ± 0.55. Over half of the women (55.1%) gave birth spontaneously and the remaining 44.9% delivered by caesarean section. Nearly one fifth (18.4%) of women had postpartum anemia. The women had median serum concentrations of 18.0 μmol/l, 1.28 mmol/l, 23.4 μmol/l, 90.1 μmol/l, 1.60 mmol/l, 34.0 μg/l and 0.81 μmol/l for Fe, Mg, Cu, Zn, Ca, Pb and Mn, respectively (Table 1).

**Table 1:** Characteristic of subjects.

	Total (*N* = 14,829)
Age, years	29.9 *±* 4.80
BMI, kg/cm*^2^*	26.2 *±* 3.11
Gestational week of delivery, weeks	38.9 *±* 1.76
Parity, times	1.41 *±* 0.55
Postpartum anemia, *N* (%)	
Yes	2,728 (18.4)
No	12,101 (81.6)
Delivery mode	
Natural birth	8,167 (55.1)
Caesarean section	6,662 (44.9)
Immediate postpartum hemorrhage	
Yes	220 (1.5)
No	14,609 (98.5)
Intrapartum injury	
Yes	148 (1.0)
No	14,659 (98.9)
Measurement time, gestational weeks	18.2 *±* 3.51
Concentrations of metal, median (interquartile)	
Iron (Fe), μmol/L	18.0 (14.1, 22.0)
Magnesium (Mg), mmol/L	1.28 (1.20, 1.41)
Copper (Cu), μmol/L	23.4 (19.0, 27.6)
Zinc (Zn), μmol/L	90.1 (79.1, 108)
Calcium (Ca), mmol/L	1.60 (1.50, 1.72)
Lead (Pb), μg/L	34.0 (24.0, 45.9)
Manganese (Mn), μmol/L^a^	0.81 (0.73, 0.89)

^a^The sample size for Mn is: *n* = 4,915.

As shown in Table 2, serum metal concentrations tended to be related by Spearman correlation analysis. Significant positive or negative correlations were observed between several (but not all) pairs of metals, while the correlations were weak or very weak (*r* = −0.165 ~ 0.331, all *p* < 0.01). A very strong positive correlation was observed between Zn and Mn (*r* = 0.846, *p* < 0.001).

**Table 2:** Spearman correlation between the serum metal concentrations.

Coefficients	Iron (Fe)	Magnesium (Mg)	Copper (Cu)	Zinc (Zn)	Calcium (Ca)	Lead (Pb)	Manganese (Mn)
Iron (Fe)	–	–	–	–	–	–	–
Magnesium (Mg)	−0.043^***^	–	–	–	–	–	–
Copper (Cu)	−0.035^***^	−0.134^**^	–	–	–	–	–
Zinc (Zn)	−0.039^***^	0.331^***^	−0.130^***^	–	–	–	–
Calcium (Ca)	0.027^**^	0.124^***^	0.156^***^	−0.165^***^	–	–	–
Lead (Pb)	0.002	0.010	0.030^***^	−0.103^***^	0.078^***^	–	–
Manganese (Mn)	0.007	−0.047^**^	−0.039**	0.846^***^	−0.007	−0.137^***^	–

^**^p < 0.01; ^***^p < 0.001.

As shown in Table 3, after adjusting for potential covariates in the Cox regression analyses, higher serum concentrations of Fe, Mg and Zn were associated with a lower risk of postpartum anemia, while a higher serum concentration of Cu was associated with a higher risk of postpartum anemia. Compared with the risk of postpartum anemia in the women whose serum concentrations of Fe were in Q1, the risk of postpartum anemia decreased by 27% in the women whose serum concentrations of Fe were in Q2 (hazard ratio [HR]: 0.73, 95% confidence interval [95% CI]: 0.65, 0.81), decreased by 35% in the women whose serum concentrations of Fe were in Q3 (HR: 0.65, 95% CI: 0.58, 0.73), decreased by 43% in the women whose serum concentrations of Fe were in Q4 (HR: 0.57, 95% CI: 0.51, 0.64), and decreased by 43% in the women whose serum concentrations of Fe were in Q5 (HR: 0.57, 95% CI: 0.50, 0.64). Similarly, higher serum concentrations of Mg were associated with decreases in the risk of postpartum anemia: 17, 33 and 33% for the women whose serum concentrations of Mg were in Q3 (HR: 0.83, 95% CI: 0.74, 0.93), Q4 (HR: 0.67, 95% CI: 0.60, 0.75) and Q5 (HR: 0.67, 95% CI: 0.60, 0.76), respectively. The women whose serum concentrations of Zn were in Q4 (HR: 0.85, 95% CI: 0.75, 0.96) and Q5 (HR: 0.82, 95% CI: 0.73, 0.93) had 15 and 18% lower risks of postpartum anemia than the women whose serum concentrations of Zn were in Q1. In contrast, compared with the women whose serum concentrations of Cu were in Q1, the women whose serum concentrations of Cu were in Q2, Q3, Q4 and Q5 had 1.15-, 1.22-, 1.31- and 1.41-fold greater risks of postpartum anemia, respectively. A small increased risk of postpartum anemia (HR:1.15, 95%CI: 1.01, 1.29) was found for women whose serum concentrations of Ca were in Q3. No significant associations were found between women’s serum concentrations of Pb and Mn and their risk of postpartum anemia.

**Table 3:** Cox regression analyses of quartile of metal concentrations and postpartum anemia.

Median (interquartile)	Quartile of metal concentrations												
	Q1		Q2			Q3			Q4			Q5	
	HR		HR	95%CI		HR	95%CI		HR	95%CI		HR	95%CI
Fe, μmol/L	11.0 (9.60, 12.3)		15.0 (14.0, 16.0)			18.0 (17.2, 19.0)			21.0 (20.3, 22.0)			26.2 (24.7, 29.0)	
Model 1	1.00		0.73	(0.65, 0.81)^***^		0.66	(0.59, 0.73)^***^		0.58	(0.52, 0.65)^***^		0.58	(0.52, 0.66)^***^
Model 2	1.00		0.73	(0.65, 0.81)^***^		0.65	(0.58, 0.73)^***^		0.57	(0.51, 0.64)^***^		0.57	(0.50, 0.64)^***^
Mg,mmol/L	1.14 (1.11, 1.16)		1.22 (1.20, 1.24)			1.29 (1.27, 1.31)			1.38 (1.35, 1.42)			1.63 (1.53, 1.80)	
Model 1	1.00		0.96	(0.86, 1.07)		0.81	(0.72, 0.91)^**^		0.68	(0.60, 0.76)^***^		0.69	(0.62, 0.78)^***^
Model 2	1.00		0.96	(0.86, 1.08)		0.83	(0.74, 0.93)^**^		0.67	(0.60, 0.75)^***^		0.67	(0.60, 0.76)^***^
Cu, μmol/L	15.0 (12.9, 16.6)		19.9 (19.0, 20.8)			23.4 (22.5, 24.2)			26.7 (25.7, 27.6)			30.7 (29.7, 32.3)	
Model 1	1.00		1.13	(0.997, 1.27)		1.18	(1.04, 1.33)^**^		1.28	(1.14, 1.44)^***^		1.40	(1.24, 1.58)^***^
Model 2	1.00		1.15	(1.02, 1.30)^*^		1.22	(1.08, 1.38)^**^		1.31	(1.16, 1.48)^***^		1.44	(1.28, 1.63)^***^
Zn, μmol/L	72.1 (68.7, 74.9)		80.9 (79.1, 83.0)			90.2 (87.6, 93.4)			103.8 (100.0, 108.1)			123.7 (117.4, 134.1)	
Model 1	1.00		1.05	(0.93, 1.17)		0.91	(0.81, 1.03)		0.87	(0.77, 0.98)^*^		0.85	(0.75, 0.96)^**^
Model 2	1.00		1.06	(0.94, 1.19)		0.91	(0.81, 1.02)		0.85	(0.75, 0.96)^**^		0.82	(0.73, 0.93)^**^
Ca, mmol/L	1.33 (1.22, 1.42)		1.53 (1.51, 1.55)			1.60 (1.58, 1.62)			1.69 (1.66, 1.72)			1.84 (1.80, 1.90)	
Model 1	1.00		0.93	(0.82, 1.05)		1.11	(0.98, 1.25)		1.08	(0.97, 1.22)		1.05	(0.93, 1.18)
Model 2	1.00		0.95	(0.84, 1.08)		1.15	(1.01, 1.29)^*^		1.12	(0.995, 1.26)		1.06	(0.95, 1.20)
Pb, μg/L	17.0 (12.1, 20.4)		25.9 (24.0, 27.8)			34.0 (32.0, 36.0)			43.0 (40.6, 45.9)			57.0 (52.0, 66.0)	
Model 1	1.10 (0.97, 1.23)		1.00	(0.89, 1.28)		1.02	(0.90, 1.14)		0.95	(0.84, 1.07)		1.00	
Model 2	1.11 (0.99, 1.25)		1.02	(0.90, 1.14)		1.04	(0.92, 1.17)		0.98	(0.87, 1.10)		1.00	
Mn, μmol/L	0.64 (0.57, 0.68)		0.75 (0.73, 0.77)			0.81 (0.80, 0.83)			0.87 (0.86, 0.89)			0.97 (0.94, 1.02)	
Model 1	1.00		1.08	(0.87, 1.32)		1.13	(0.92, 1.39)		1.00	(0.81, 1.23)		1.05	(0.85, 1.30)
Model 2	1.00		1.08	(0.88, 1.33)		1.11	(0.90, 1.36)		0.99	(0.80, 1.23)		1.04	(0.84, 1.28)

Model 1: univariate model. Model 2: adjusted for age, BMI, parity, gestational week at delivery, parity, delivery mode, immediate postpartum hemorrhage, and intrapartum injury. *p<0.05; **p<0.01; ***p<0.001.

**Figure 1:**
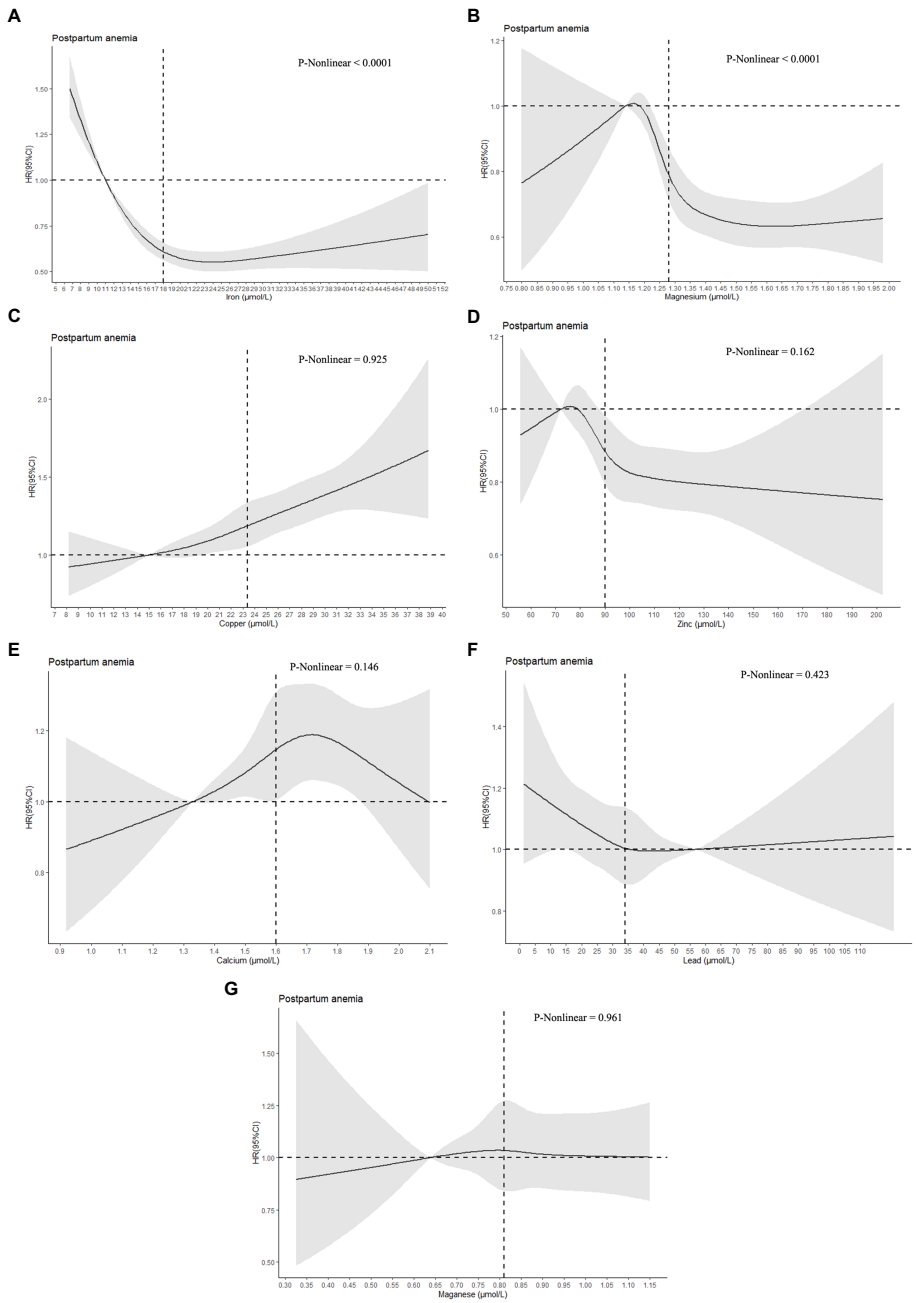
Dose–response associations between serum concentrations of metals and postpartum anemia. Analyses were performed by restricted cubic spline regressions (5 knots). The median concentrations of iron, magnesium, copper, zinc, calcium, and magnanese in Q1 were used as the reference values (HR = 1.00) for analyses of these species, while the median concentration of Pb in Q5 was assigned as the reference value (HR = 1.00) for this species. The vertical lines represent the median metal concentrations. Parts A-G represent the dose-response associations of iron, magnesium, copper, zinc, calcium, lead, and magnanese with postpartum anemia.

Restricted cubic spline regression was performed to investigate dose–response associations between serum concentrations of metals and postpartum anemia. The median concentrations of Fe, Mg, Cu, Zn, Ca and Mn in Q1 were used as the reference values for analyses of these species, while the median concentration of Pb in Q5 was assigned as the reference value for this species. As shown in Figure 1, an L-shaped curve association was observed between serum concentrations of Fe and postpartum anemia, with a significant nonlinearity (*p* < 0.0001). Increasing serum concentrations of Fe were associated with a lower risk of postpartum anemia up to a concentration of 18.4 μmol/l, with this protective association decreasing at serum concentrations of Fe greater than 22.0 μmol/l. Similar L-shaped curve associations were found between serum concentrations of Mg and Zn and the risk of postpartum anemia. The risk of postpartum anemia decreased rapidly as serum concentrations of Mg and Zn increased from 1.20 to 1.40 μmol/l and from 80 to 100 μmol/l, respectively, but thereafter decreased less rapidly. Significant nonlinearity was found for Mg (*p* < 0.0001) but not for Zn (*p* = 0.162). In contrast, the risk of postpartum anemia increased as serum concentrations of Cu increased (*p* for nonlinearity = 0.925). An inverse U-shaped association was observed between serum concentrations of Ca and the risk of postpartum anemia, with no significant nonlinearity detected (*p* = 0.146). No significant associations were observed between serum concentrations of Pb and Mn and the risk of postpartum anemia.

We observed that higher quintiles of Cu were associated with higher HR of postpartum anemia, whereas higher quintiles of Fe, Mg, and Zn were associated with lower HR of postpartum anemia (Table 3). We aimed to investigate potential protective associations of the combination of serum metal concentrations and postpartum anemia. To avoid the potential confounding influence of Cu, inverse Cu was introduced instead of Cu in the combination with other metals (Fe, Mg, and Zn). As shown in Table 4, compared with high serum concentrations of Fe alone, high serum concentrations of Mg, Zn and/or inverse Cu in combination with high serum concentrations of Fe were associated with a lower risk of postpartum anemia. HRs for postpartum anemia ranged from 0.31 to 0.37 when high serum concentrations of Mg, Zn or inverse Cu occurred in combination with a high serum concentration of Fe, compared with a HR of 0.51 for postpartum anemia when there was only a high serum concentration of Fe. HRs ranged from 0.21 to 0.23 when high serum concentrations of two of Mg, Zn and inverse Cu occurred in combination with high serum concentrations of Fe, compared with an HR of 0.46 when there was only a high serum concentration of Fe. The HR for postpartum anemia was 0.10 when high serum concentrations of Mg, Zn and inverse Cu occurred in combination with a high serum concentrations of Fe, compared with an HR of 0.44 for postpartum anemia when there was only a high serum concentration of Fe.

**Table 4:** Cox regression analyses of combination of different metal concentrations and postpartum anemia.

Co-existence of bottom quintile groups of different metals	Bottom groups		Co-existence of top group of different division of metal combinations							
	*N*	Reference	5 division^a^		10 division^a^		15 division^a^		20 division^a^	
			*N*	HR (95%CI)	*N*	HR (95%CI)	*N*	HR (95%CI)	*N*	HR (95%CI)
Fe	n^b^	1.00	2,958	0.57 (0.50, 0.64)^***^	1,484	0.51 (0.44, 0.60)^***^	1,042	0.46 (0.39, 0.56)^***^	776	0.44 (0.36, 0.55)^***^
Fe + Mg	623	1.00	–	–	522	0.31 (0.24, 0.41)^***^	–	–	–	–
Fe + Zn	551	1.00	–	–	532	0.36 (0.27, 0.49)^***^	–	–	–	–
Fe + inverse Cu^c^	638	1.00	–	–	601	0.37 (0.29, 0.48)^***^	–	–	–	–
Fe + Mg + Zn	169	1.00	–	–	–	–	226	0.21 (0.13, 0.33)^***^	–	–
Fe + Mg + inverse Cu^c^	135	1.00	–	–	–	–	198	0.21 (0.13, 0.33)^***^	–	–
Fe + Zn + inverse Cu^c^	104	1.00	–	–	–	–	175	0.23 (0.13, 0.43)^***^	–	–
Fe + Mg + Zn + inverse Cu^c^	36	1.00	–	–	–	–	–	–	99	0.10 (0.04, 0.25)^***^

^a^Divisions means the groups of the numbers of separations of the serum Fe concentrations or the numbers of the groups of the combine calculation of different metals. ^b^The sample in the bottom groups of different divisions of Fe were 2,961 for 5 division; 1,506 for 10 division; 989 for 15 division; and 747 for 20division. ^c^For inverse Cu, subjects with the highest serum Cu concentrations were assigned as the bottom quintile group (Q1), while those with lowest serum Cu concentrations were in the top quintile group (Q5). Model 1: univariate model. Model 2: adjusted for age, BMI, parity, gestational week at delivery, parity, delivery mode, immediate postpartum hemorrhage, and intrapartum injury.

## Discussion

In this large retrospective cohort study, higher serum concentrations of Fe, Mg and Zn and lower serum concentrations of Cu in pregnancy contributed to lower risk of postpartum anemia. The association of the risk of postpartum anemia with serum concentrations of Fe, Mg and Zn decreased rapidly as serum concentrations of Fe increased from 6.5 to 22.0 μmol/l, serum concentrations of Mg increased from 1.20 to 1.40 μmol/l and as serum concentrations of Zn increased from 80 to 100 μmol/l, but the curves soften with further increment. Increased serum concentrations of Cu had a linear association with an increased risk of postpartum anemia. Compared with high serum concentrations of Fe alone, the coexistence of high serum concentrations of Fe with high serum concentrations of Mg and Zn and with low serum concentrations of Cu was associated with a lower risk of postpartum anemia.

Fe is an essential micronutrient for maintaining hemoglobin concentrations, so adequate serum concentrations of Fe are needed to prevent anemia. Oral or IV Fe supplementation increases hemoglobin concentration and thus prevents and treats all forms of anemia (6, 8–10). However, Fe overload increases the level of oxidative stress in the human body (20) and increases the risk of developing other diseases during pregnancy, such as GDM (12) and preeclampsia (21). Moreover, long-term periconceptional Fe supplementation of more than 30 mg/d was associated with increased GDM risk in a large prospective cohort of pregnant Chinese women (12). Thus, there is a need to optimize the use of Fe supplements to prevent postpartum anemia while avoiding the risk of predisposing women to adverse outcomes and the development of other diseases. Further studies are thus needed on Fe supplementation and maintaining appropriate serum concentration of Fe. In the current study, we found that the risk of postpartum anemia was decreased in women with serum concentrations of Fe of 6.5 to 22.0 μmol/l. This result needs to be validated in future high-quality studies using comprehensive Fe indicators.

There have been fewer studies on the relationship between serum concentrations of Zn and anemia than on the relationship between serum concentrations of Fe anemia, especially during pregnancy; however, most studies have indicated that a certain serum concentration of Zn protects against anemia. A lower serum concentration of Zn was associated with risk of anemia and Fe deficiency low concentrations of hemoglobin during pregnancy in a study of 1,185 pregnant Chinese women (22). A study of pregnant women in rural Bangladesh also showed found an association between low serum concentrations of Zn and low concentrations of hemoglobin, despite minimal Fe deficiency (23). Higher plasma or serum concentrations of Zn have also been found to be associated with higher concentrations of hemoglobin in non-pregnant populations (16, 24, 25). In addition, Zn supplementation was found to improve anemia status in certain populations, such as infants (26). It was suggested that Zn plays a role in modulating Fe metabolism by regulating the expression of divalent metal-ion transporter-1 and ferroportin, thereby affecting Fe uptake and transcellular transport (18).

There have been a few low-quality studies on the association between serum concentrations of Mg and postpartum anemia. In a cross-sectional study of 180 pregnant Sudanese women, serum concentrations of Mg were positively associated with concentrations of hemoglobin, and women with anemia had significantly lower concentrations of Mg than women without anemia (27). In older Chinese individuals, serum concentrations of Mg were found to mediate the associations between diet and development of anemia (17). In another study of 2,849 Chinese adults, a higher intake Mg was found to be associated with a lower risk of anemia and this association was not modified by serum concentrations of ferritin (15). Thus, current evidence partly supports our observations that higher serum concentrations of Mg contribute to a lower risk of postpartum anemia. Higher serum concentrations of Mg in pregnancy may also prevent the occurrence of gestational hypertensive disorders such as pre-eclampsia, which make women prone to bleeding (28). Magnesium sulfate is commonly used in the treatment of these diseases, and the dose should be carefully controlled to prevent Mg poisoning (29). In this study, the risk of postpartum anemia was decreased in women with serum concentrations of Mg ranging from 1.20 to 1.40 μmol/l. These results serves as reference data that needs to be validated in future studies.

The association between serum concentrations of Cu and postpartum anemia appears more complex. Cu deficiency causes anemia and leads to lower plasma and brain concentrations of Fe in rats (30). In a cross-sectional study, serum concentrations of Cu and hemoglobin were found to have an negative association with anemia and the ratio of Cu to Fe was higher among pregnant women with anemia than among pregnant women without anemia (22). In children, higher serum concentrations of Cu were found among those with iron deficiency anemia who also exhibited decreased Fe absorption and deficient hematological parameters (19). It is possible that U-shaped associations between serum concentrations of Cu and postpartum anemia may exist as similar associations have been previously found for unexplained anemia (31) and new-onset hypertension (32). In the current study, high serum concentrations of Cu was independently and positively associated with the risk of postpartum anemia. This may be because the pregnant women in our study were less likely to be Cu deficient than those in other studies. Thus, there needs to be more examination on the risk of Cu overload during pregnancy and how this risk can be mitigated. Our results show that high serum concentrations of Cu should be avoided to prevent postpartum anemia in women.

We also found that a deficiency of Fe in combination with an imbalance in one or more other metal nutrients (Zn deficiency, Mg deficiency and/or Cu overload) was associated with a higher risk of postpartum anemia than a deficiency of Fe alone. Our results are supported by another study that found that adults with the lowest risk of anemia in were those who also had the highest intake of Mg and Fe. However, studies of the effects in pregnant women of multiple supplementations with Fe or Fe–folic acid (IFA) and/or multiple micronutrients (MM) have yielded inconsistent results. In a study of1,813 pregnant Vietnamese women, MM and IFA supplementation resulted in increased Fe stores but did not have an effect on anemia (33). In a study among pregnant women in rural Nepal, supplementation with IFA + Zn or IFA + Zn + MM did not provide additional benefits in improving maternal hematologic status compared with IFA supplementation alone (34). In a study of pregnant women in semirural Mexico, MM supplementation slightly reduced concentrations of hemoglobin compared with Fe supplementation (35). In a study among women with postpartum anemia who were treated with oral Fe supplements, Zn supplementation had a negative but transient influence on hematological status; however, this finding was not clinically significant (36).

The abovementioned findings may indicate that women with simultaneous deficiencies of Fe and other micronutrients may be less likely to be involved in randomized controlled trials (RCTs) with a relatively small sample size than women without simultaneous deficiencies of Fe and other micronutrients. Moreover, over-supplementation of several nutrients *via* MM may not provide obvious additional benefits for individuals who do not have existing deficiencies and may even have negative effects. In the current study, we identified that women with deficiencies in Fe and concomitant imbalances in other metal nutrient (a Zn deficiency, a Mg deficiency and/or a Cu overload) had a higher risk of postpartum anemia than those who were only deficient in Fe, which highlights the need to treat women with such multiple metal -nutrient deficiencies to prevent postpartum anemia. Large RCTs are needed to focus on these special populations and elaborate associations between metal nutrient imbalance and postpartum anemia.

### Strength and limitations

This study had several strengths. First, serum concentrations of metals were measured during pregnancy before delivery, and the exclusion criteria meant that we included only women whose serum concentrations of metals were measured before 28 weeks of gestation. This helped to ensure the temporal sequence of events and avoid possible causal inversions. Second, the large sample size enabled us to divide the population into different groups and thus obtain more accurate results and narrower 95% CIs for HRs than would have been possible otherwise. Third, the use of restricted cubic spline regression and investigation of the simultaneous deficiency of Fe and several other metals provided comprehensive results on the associations of metal nutrients with postpartum anemia.

However, there were also several limitations to this study. First, the data were collected from a single obstetric center in Southern China. Although this center is the largest obstetric center in its city and covers a large population, there may have been selection bias, so further studies based on more representative populations are needed. Second, serum concentrations of metals were measured only once; no data for multiple time points were available. Thus, we were unable to explore the associations between serum metal concentrations over time and postpartum anemia. Third, data on dietary metal intake were not available in this study. Thus we were unable to examine the associations between dietary metal intake and postpartum anemia, which should be further investigated by high-quality studies in the future. Four, the use of oral iron supplements during pregnancy was not collected during the regular obstetric check-up, and we could not obtain related data and could not make up the missing of these data due to the retrospective design. Therefore, it was not included in the statistics and be adjusted for. However, the use of iron supplementation may have tended to underestimate rather than overestimate the associations found in our study. Finally, aside from serum concentrations of ferritin, transferrin, soluble transferrin receptor and hepcidin are also important indicators for measuring Fe metabolism status. Unfortunately, these were not measured during regular obstetric check-ups, meaning we were unable to explore more comprehensive associations between iron metabolism indicators and postpartum anemia, and possible interactions with other metals indicators.

## Conclusion

In this large retrospective cohort study of Chinese women with singleton pregnancies, we found that higher serum concentrations of Fe, Mg and Zn before 28 weeks of gestation were associated with lower risk of postpartum anemia. The risk of postpartum anemia showed large and significant decreases as serum concentrations of Fe, Mg and Zn increased within a certain range and then the risk decreased as the serum concentrations of these species increased beyond this range. In contrast, serum concentrations of Cu were positively associated with an increased risk of postpartum anemia. Women who had high serum concentrations of Fe in combination with high serum concentrations of Mg and Zn and low serum concentrations of Cu exhibited a lower risk of postpartum anemia than women who only had high serum concentrations of Fe. Our study had emphasized the importance of addressing imbalances of multiple metal nutrients as a strategy to prevent postpartum anemia. More high-quality studies are needed to determine a comprehensive strategy for managing serum concentrations of metal nutrients during pregnancy to prevent postpartum anemia.

## Data availability statement

The raw data supporting the conclusions of this article will be made available by the authors, without undue reservation.

## Ethics statement

The studies involving human participants were reviewed and approved by the ethics committee of the Affiliated Foshan Maternity and Child Healthcare Hospital, Southern Medical University. Written informed consent for participation was not required for this study in accordance with the national legislation and the institutional requirements.

## Author contributions

G-dC, H-lL, and Z-pL devised the idea and designed the study. G-dC, T-tP, P-sL, Z-xZ, X-yG, H-yW, D-xL, and D-zF contributed to the primary data collection. G-dC and T-tP re-examined the data and analysis the data. G-dC and T-tP wrote the original draft, which was revised by H-lL and Z-pL H-lL and Z-pL supervised the study and administered the project. All authors contributed to the article and approved the submitted version.

## Funding

This work was supported by National Natural Science Foundation of China (grant numbers 82103855, G-dC), Basic and Applied Basic Research Foundation of Guangdong Province (grant numbers 2019A1515110163, G-dC) and the Foundation of Bureau of Science and Technology of Foshan City (grant numbers 2220001004104, G-dC). The funding sponsors had no role in the design of the study; in the collection, analyses, or interpretation of data; in the writing of the manuscript, and in the decision to publish the results.

## Conflict of interest

The authors declare that the research was conducted in the absence of any commercial or financial relationships that could be construed as a potential conflict of interest.

## Publisher’s note

All claims expressed in this article are solely those of the authors and do not necessarily represent those of their affiliated organizations, or those of the publisher, the editors and the reviewers. Any product that may be evaluated in this article, or claim that may be made by its manufacturer, is not guaranteed or endorsed by the publisher.

## Footnotes

^1^ https://www.r-project.org/
